# The Role of Vitamin D in Fertility and during Pregnancy and Lactation: A Review of Clinical Data

**DOI:** 10.3390/ijerph15102241

**Published:** 2018-10-12

**Authors:** Stefan Pilz, Armin Zittermann, Rima Obeid, Andreas Hahn, Pawel Pludowski, Christian Trummer, Elisabeth Lerchbaum, Faustino R. Pérez-López, Spyridon N. Karras, Winfried März

**Affiliations:** 1 Department of Internal Medicine, Division of Endocrinology and Diabetology, Medical University of Graz, Auenbruggerplatz 15, 8036 Graz, Austria; christian.trummer@medunigraz.at (C.T.); elisabeth.lerchbaum@medunigraz.at (E.L.); 2 Clinic for Thoracic and Cardiovascular Surgery, Heart Center North Rhine-Westfalia, Ruhr University Bochum, Georgstraße 11, D-32545 Bad Oeynhausen, Germany; azittermann@hdz-nrw.de; 3 Department of Clinical Chemistry and Laboratory Medicine, University Hospital of the Saarland, Building 57, D-66421 Homburg/Saar, Germany; rima.obeid@uks.eu; 4 Institute of Food Science and Human Nutrition, Leibniz University Hannover, Am Kleinen Felde 30, D-30167 Hannover, Germany; hahn@nutrition.uni-hannover.de; 5 Department of Biochemistry, Radioimmunology and Experimental Medicine, The Children’s Memorial Health Institute, Aleja Dzieci Polskich 20 Str, 04730 Warsaw, Poland; pludowski@yahoo.com; 6 Department of Obstetrics and Gynecology, University of Zaragoza, Faculty of Medicine, Lozano-Blesa University Hospital, Domingo Miral s/n, 50009 Zaragoza, Spain; faustino.perez@unizar.es; 7 Division of Endocrinology and Metabolism, First Department of Internal Medicine, Medical School, Aristotle University of Thessaloniki, AHEPA Hospital, 55535 Thessaloniki, Greece; karraspiros@yahoo.gr; 8 Medical Clinic V (Nephrology, Hypertensiology, Rheumatology, Endocrinology, Diabetology), Medical Faculty Mannheim, University of Heidelberg, Theodor- Kutzer-Ufer 1-3, D-68167 Mannheim, Germany; 9 Clinical Institute of Medical and Chemical Laboratory Diagnostics, Medical University of Graz, Auenbruggerplatz 15, 8036 Graz, Austria; 10 SYNLAB Academy, SYNLAB Holding Deutschland GmbH, P5, 7, D-68161 Mannheim and Augsburg, Germany; winfried.maerz@synlab.com

**Keywords:** vitamin D, gestational diabetes, pre-eclampsia, breast milk, vitamin D binding protein, DBP, brain, autism, 25-hydroxyvitamin D

## Abstract

Vitamin D deficiency is common and there exists a huge gap between recommended dietary vitamin D intakes and the poor vitamin D supply in the general population. While vitamin D is important for musculoskeletal health, there are accumulating data suggesting that vitamin D may also be important for fertility, pregnancy outcomes and lactation. Significant changes in vitamin D metabolism during pregnancy such as increased production of the “active vitamin D hormone” calcitriol support the important role of vitamin D in this setting. Observational studies show that vitamin D deficiency is a risk marker for reduced fertility and various adverse pregnancy outcomes and is associated with a low vitamin D content of breast milk. Meta-analyses of randomized controlled trials (RCTs) document that physiological vitamin D supplementation during pregnancy is safe and improves vitamin D and calcium status, thereby protecting skeletal health. Although certain RCTs and/or meta-analyses reported some other beneficial effects, it is still not clear whether vitamin D supplementation improves fertility or decreases the risk of adverse pregnancy outcomes such as low birth weight, pre-eclampsia and neonatal mortality, or reduces wheeze/asthma in the infants. Nevertheless, vitamin D supplementation in pregnant women is frequently required to achieve a sufficient vitamin D status as recommended by nutritional vitamin D guidelines. In this review, we provide an overview of systematic reviews, meta-analyses and large trials reporting clinical data on the role of vitamin D for fertility, pregnancy and lactation.

## 1. Introduction

Vitamin D deficiency is considered a worldwide public health problem, in particular because in most countries, large parts of the general population do not meet the dietary vitamin D requirements as recommended by nutritional vitamin D guidelines [1,2,3,4,5,6,7,8,9,10]. Vitamin D is important for musculoskeletal health and, historically, is known to be effective for the prevention and treatment of rickets and osteomalacia, and may also reduce fractures and falls in the elderly [11,12,13,14]. Several observational studies have shown that a poor vitamin D status is associated with various extra-skeletal diseases such as cardiovascular and metabolic diseases, cancer, autoimmune and neurological diseases [14,15]. By contrast, randomized controlled trials (RCTs) have, in the majority, failed to show clinically relevant effects of vitamin D supplementation on these outcomes [16,17,18]. Therefore, it has been suggested that vitamin D deficiency may be rather a risk marker for ill health than a causal factor for many diseases [19]. Consequently, there is still an ongoing scientific controversy on potential extraskeletal effects of vitamin D that is beyond the scope of the present review. It must, however, be underlined that meta-analyses of RCTs support the notion that vitamin D supplementation may reduce the risk of mortality, infections, asthma exacerbations and some pregnancy outcomes, although these data have certain limitations and it might be premature to clearly establish or claim causality [20,21,22,23,24,25,26,27,28,29,30,31].

### Literature Review

In this review, we provide an overview of clinical data regarding the potential role of vitamin D in fertility and during pregnancy and lactation. This work is based on a literature search in Pubmed until 12 August 2018 using the search terms “vitamin D” plus “systematic review” or “meta-analysis” plus “fertility”, “pregnancy”, “lactation”, “breastfeeding” or “breast milk”. References from selected articles and personal reference lists were also used to expand the search. We included systematic reviews and meta-analyses of both, observational studies and RCTs. We also discuss some single clinical studies if we consider them to be of relevance because they were still not included in meta-analyses as they were published afterwards or if there are no meta-analyses available on particular outcomes. We did not restrict our search to cover specific outcomes but rather aimed to include all available outcomes regarding fertility as well as pregnancy and lactation because the purpose of this review is to provide an up to date overview of all clinical data on these issues. We refer the reader to other publications regarding physiologic mechanisms underlying the proposed effects of vitamin D on fertility, pregnancy and lactation [31,32,33,34,35,36,37,38,39,40,41,42].

After an introduction on vitamin D metabolism with a specific focus on changes during pregnancy, we summarize, in separate sections, clinical data with particular attention on meta-analyses of RCTs on the potential effects of vitamin D on fertility, pregnancy and lactation. Finally, we give some guidance and recommendations regarding the clinical use of vitamin D treatment and present our conclusions.

## 2. Vitamin D Metabolism

### 2.1. General Vitamin D Metabolism

Vitamin D exists in two main isoforms, vitamin D3 (cholecalciferol) and vitamin D2 (ergocalciferol) [32]. Vitamin D3 is either derived from ultraviolet-B (UV-B) induced production from its precursor 7-dehydrocholesterol in the skin or from intake of foods such as fatty fish, cod liver oil or egg yolk, whereas vitamin D2 is derived from intake of fungal sources such as mushrooms and yeast. As vitamin D3 and D2 have a similar metabolism we do not differentiate between them if not otherwise stated and refer to vitamin D without distinguishing D2 and D3 throughout this manuscript. In addition to synthesis in the skin and intake by natural foods, vitamin D supply also comes from supplement intake and vitamin D-fortified foods. Several countries such as the US, Finland, Canada and India have already introduced systematic vitamin D food fortification of e.g., milk products [3]. According to a rough estimate, about 80% of vitamin D supply is derived from endogenous production in the skin whereas only about 20% of vitamin D supply is derived from oral intake [33]. There exists, however, a significant individual and seasonal variation in vitamin D supply and vitamin D status.

In humans, vitamin D is converted to 25-hydroxyvitamin D (25(OH)D) by different 25-hydroxylase enzymes in the liver. Serum 25(OH)D has a traced half-life of approximately 2 to 3 weeks and is used for the classification of vitamin D status because it best reflects vitamin D supply from all different sources. In the circulation, about 85 to 90% of 25(OH)D is bound to vitamin D-binding protein (DBP), 10 to 15% to albumin and only less than 1% of serum 25(OH)D is unbound (i.e., free) [34]. At present, vitamin D status classification is based on total 25(OH)D, i.e., the sum of bound and unbound 25(OH)D, but there may be a value for measuring free 25(OH)D because according to the “free hormone hypothesis” only the unbound fraction can cross the cell membrane to exert its effects within the cells [34]. Some tissues such as the kidneys, parathyroid glands and placenta are, however, able to take up DBP-bound 25(OH)D by the megalin/cubilin complex. Therefore, as well as due to some knowledge gaps, the biological role of free 25(OH)D and other free vitamin D metabolites still needs to be further evaluated. While 25(OH)D is measured to assess vitamin D status, a further hydroxylation step of 25(OH)D is required to produce 1,25-dihydroxyvitamin D (1,25(OH)2D), the so-called active vitamin D hormone or calcitriol, that has the highest affinity for the almost ubiquitously expressed vitamin D receptor (VDR). Serum 1,25(OH)2D is mainly derived from the 1-alpha-hydroxylase catalyzed conversion of 25(OH)D to 1,25(OH)2D in the kidneys, but several extra-renal tissues are also able to convert 25(OH)D to 1,25(OH)2D on a local/tissue level. Renal 1-alpha-hydroxylase activity is tightly regulated by parameters of mineral metabolism and is e.g., stimulated by parathyroid hormone (PTH) and inhibited by fibroblast-growth factor-23 (FGF-23) whereas extra-renal 1,25(OH)2D production is highly substrate-dependent and differentially regulated. The biological effects of 1,25(OH)2D are mediated by binding to the VDR with downstream regulation of the expression of hundreds of genes. Therefore, 1,25(OH)2D functions as a classic steroid hormone such as thyroid or sex hormones. Degradation of vitamin D metabolites is initiated by 24-hydroxylation leading to the formation of calcitroic acid after several hydroxylation and oxidation steps. Calcitroic acid is finally excreted in the bile and urine. For more detailed descriptions of basic vitamin D metabolism we refer the reader to other excellent reviews on this topic [33,34].

Vitamin D guidelines from major health authorities raised recommendations for vitamin D intakes (i.e., intakes by diet plus supplements) that are based on the assumption of minimal or no sun exposure [4,5,6,7,8]. The Institute of Medicine (IOM) termed vitamin D intakes that meet the requirements of 50% and 97.5% of the population, as the estimated average requirement (EAR) and the recommended dietary allowance (RDA), respectively (5). The process of formulating these dietary reference intakes is based on the assumption that serum 25(OH)D concentrations are a biomarker of vitamin D status and intakes. In general, the cause and effect relationship of vitamin D and musculoskeletal (and not extra-skeletal) health outcomes was used to inform dietary vitamin D requirements. Dose response relationships of serum 25(OH)D and musculoskeletal health outcomes were used to establish “EAR-like” and “RDA-like” serum 25(OH)D concentrations. Then, meta-regression analyses of vitamin D RCTs in winter (with no endogenous vitamin D synthesis in the skin) were used to calculate the required vitamin D intake doses to achieve the “EAR-like” (i.e., 40 nmol/L; multiply by 2.496 to convert ng/ml to nmol/L) and “RDA-like” (i.e., 50 nmol/L) serum 25(OH)D concentrations. Similar approaches have been made by other major health agencies and are listed in Table 1 [6,7,8]). There are several limitations surrounding these guidelines, but it should be considered that advanced statistical methods, i.e., individual participant data (IPD) meta-analyses, suggest that current RDAs or RDA-like intakes, may underestimate the actual intake requirements to achieve certain serum 25(OH)D concentrations in 97.5% of the population [1,2]. This notion is based on the fact that guidelines such as those of the IOM report used conventional meta-regression analyses that are based on group (aggregate) data and not IPD and, therefore, do not sufficiently capture between individual variability [1,2]. In this context, Cashman et al. have shown, by using the IPD approach, that an overall vitamin D intake (i.e., supplements plus diet) of about 1000 IU (40 IU equals 1 μg) per day is required to achieve a serum 25(OH)D concentration of ≥50 nmol/L in 97.5% of the population [1,2]. It was further calculated that an intake of 400 IU (10 μg) is required to achieve ≥25 nmol/L in 97.5% of the population. It is important to note that general populations do not meet vitamin D requirements as, for example, in Europe serum 25(OH)D concentrations <30 nmol/L and <50 nmol/L are detected in 13.0% and 40.4% of the population [9].

### 2.2. Vitamin D Metabolism during Pregnancy

Some aspects of vitamin D metabolism are of physiologic relevance in pregnancy [35,36,37,38,39,40,41,42]. Compared to non-pregnant women, there is a significant increase in 1,25(OH)2D concentrations, with a 2-fold increase in in the first trimester of pregnancy and a further rise to a 2- to 3-fold increase during the course of pregnancy and a rapid decline after delivery [35]. The kidney is the major production site of serum 1,25(OH)2D, but given that PTH concentrations are lower in pregnant compared to non-pregnant women, there are still some knowledge gaps regarding the regulation of serum 1,25(OH)2D concentrations in pregnancy [40]. It has been hypothesized that apart from proximal tubular cells, 1,25(OH)2D might also be produced by activated immune cells such as macrophages in the kidney. Other hormones such as e.g., PTH-related peptide, might also play a role in the regulation of serum 1,25(OH)2D concentrations in pregnancy. Of note is also that the positive correlation between serum 1,25(OH)2D and 25(OH)D concentrations is stronger in pregnant compared to non-pregnant women suggesting that 1,25(OH)2D synthesis is more substrate dependent, i.e., more determined by serum 25(OH)D concentrations, in pregnancy [35]. The placenta is also producing 1,25(OH)2D on a local/tissue level without significantly contributing to circulating serum 1,25(OH)2D concentrations.

Several observational studies reported an increase of DBP during pregnancy with a peak of approximately 40–50% higher serum DBP concentrations compared to non-pregnant women at the beginning of the third trimester and a decline at term [38]. The liver is usually the major production site of DBP, but given that human placental trophoblasts express DBP on their cell surface, it has been speculated that the rise in DBP concentrations might be partially the result of the high turnover rate of trophoblasts.

Despite major changes in vitamin D metabolism during pregnancy such as increased 1,25(OH)2D production there are no established changes in total serum 25(OH)D concentrations in pregnant compared to non-pregnant women, although some studies suggest that serum 25(OH)D might be lower in pregnancy [35,36,37,38]. It is not clearly established but there seems to be a modest decline in free 25(OH)D during pregnancy that may at least in part be explained by the increase in serum DBP concentrations [37]. One major point in pregnancy is that the fetus is totally dependent on the mother’s vitamin D status, which explains the very high correlation of mother and cord blood 25(OH)D concentrations. While 25(OH)D crosses the placenta, 1,25(OH)2D does not but is produced by the fetal kidneys [41]. In general, cord blood 25(OH)D concentrations are about 50 to 80% of serum 25(OH)D concentrations of the mother [39]. This underscores the importance of adequate vitamin D supply to pregnant women.

From a physiological point of view the vitamin D system is critical for bone and calcium (mineral) homeostasis, and pregnancy as well as lactation are settings that require an adequate vitamin D status to avoid disturbances in bone and mineral metabolism [41]. Importantly, vitamin D supplementation can prevent neonatal hypocalcemia, that may result in softening of bones (e.g., craniotabes and various pathologies of rickets) and, in severe cases, to seizures and dilated cardiomyopathy [8,9]. While many questions remain open regarding the physiologic role of vitamin D for calcium homeostasis during pregnancy and lactation, there are accumulating data suggesting that in pregnancy, the role of the vitamin D system becomes particularly important for immunomodulation of the maternal-fetal interface [35,36,41,42,43]. Other functions of vitamin D may be stimulation of sex hormone secretion, implantation/placentation and respiratory maturation [35,36,41,42,43]. Vitamin D may also be important for prevention of pre-eclampsia by e.g., stabilizing the endothelium through non-genomic mechanisms [35]. Furthermore, vitamin D may induce epigenetic changes and exert many other relevant effects owing to the expression of VDRs and enzymes of vitamin D metabolism throughout the reproductive tracts of men and women [35,36,41,42,43]. A review of mechanistic effects of vitamin D is, however, beyond the scope of the present work and we refer to reader to other excellent articles on this topic [32,35,36,38,41,42,43].

## 3. Fertility

According to a popular, but not universally accepted, hypothesis there was an evolutionary pressure to develop a lighter skin in order to optimize UV-B induced vitamin D synthesis in the skin [44]. Prevention of rickets and thus avoiding narrowing of the pelvis with obstructive childbirth might be a hypothetical explanation for the advantage of having a light skin. Apart from this, several studies and reviews have highlighted the potential role of vitamin D for female fertility today [45,46,47,48,49,50,51]. Interestingly, in northern countries there is a seasonal variation in pregnancy rates with a peak in summer and autumn, i.e., the seasons with the highest serum 25(OH)D concentrations [46]. Findings from a systematic review and meta-analysis including 11 studies with 2700 women on vitamin D status and outcomes of assisted reproductive treatment (ART) are in line with this [51]. Compared to women with deficient or insufficient vitamin D status, women with replete vitamin D status had more live births (odds ratio (OR): 1.33; 95% confidence interval (CI): 1.08 to 1.65), more positive pregnancy tests (OR: 1.34; 95% CI: 1.04 to 1.73) and more clinical pregnancies (OR: 1.46; 95% CI: 1.05 to 2.02), whereas there was no association between miscarriage and vitamin D status [51]. Other meta-analyses on vitamin D status and ART outcomes revealed similar findings [52,53]. Vitamin D RCTs specifically designed to assess outcomes of ART are, however, still missing. Interestingly, one meta-analysis documented that early spontaneous pregnancy loss was significantly increased in women with serum 25(OH)D concentrations below 50 nmol/L compared to those with higher 25(OH)D levels (relative risk (RR): 2.24; 95% CI: 1.15 to 4.37) [54,55,56]. In line with this, a study in 1191 women with previous pregnancy losses showed that women with serum 25(OH)D concentrations ≥75 nmol/L preconception were more likely to achieve clinical pregnancy (adjusted RR: 1.10; 95% CI: 1.01 to 1.20) and livebirth (RR: 1.15; 95% CI: 1.02 to 1.19) compared to those with lower serum 25(OH)D concentrations [57,58]. In the same study, a subgroup analysis of women who achieved pregnancy showed that preconception serum 25(OH)D was associated with a reduced risk of pregnancy loss (RR per 25 nmol/L: 0.88; 95% CI: 0.77 to 0.99) whereas serum 25(OH)D at 8 weeks of pregnancy was not [57,58]. Some, but not all observational studies reported that serum 25(OH)D concentrations are positively associated with markers of ovarian reserve such as anti-Müllerian hormone (AMH) but further data are needed on this topic [47,48,49]. Polycystic ovary syndrome (PCOS) and endometriosis, which are both associated with fertility problems, have also been associated with vitamin D deficiency in some but not all observational studies [46,50]. Importantly, there are also some papers about vitamin D RCTs in women with PCOS published that have, however, only inconsistently reported some beneficial effects of vitamin D supplementation on endocrine, metabolic and fertility aspects [50,59]. Many of these studies were limited by relatively small sample sizes and did not account for multiple testing problems [50,59]. Therefore, to date, no final conclusion can be drawn regarding potentially beneficial clinical effects of vitamin D on PCOS and its related pathologies [50,59].

Apart from female fertility, there are also accumulating data on the role of vitamin D in male fertility [60,61,62,63,64]. Observational studies reported that vitamin D deficiency is associated with low serum testosterone concentrations but RCTs have, by the majority, failed to show an effect of vitamin D supplementation on testosterone status [62,63]. Similarly, vitamin D deficiency has been associated with poor semen quality in observational studies, whereas RCTs did not show improvements following vitamin D supplementation [63].

## 4. Pregnancy Outcomes

### 4.1. Observational Studies

Several investigations have shown that low serum 25(OH)D concentrations are associated with some adverse neonatal and pregnancy outcomes [65,66,67,68]. In this context, several reviews and meta-analyses support the notion that low serum 25(OH)D concentrations are a risk factor for hypertensive disorders in pregnancy such as pre-eclampsia, and for gestational diabetes mellitus [69,70,71,72,73,74,75,76,77,78]. Moreover, in the majority of the published meta-analyses low serum 25(OH)D concentration in pregnant women are associated with an increased risk of their children for childhood asthma, wheeze, respiratory tract infections, allergic rhinitis, and eczema [79,80,81,82,83,84,85]. Some studies suggest that vitamin D status could be important for brain development, cognitive function and psychological function and may reduce the risk of autism spectrum disorders, but the data are inconclusive [86,87,88,89]. Importantly, meta-analyses of observational studies documented that low serum 25(OH)D concentrations in pregnancy are associated with increased risk of small for gestational age (SGA) and preterm birth [90,91,92]. Some meta-analyses have also shown that vitamin D deficiency is associated with postpartum depression [93,94,95,96]. Other outcomes such as fetal bone growth or neonatal lung maturation may also be associated with vitamin D status, but more data are needed on these topics [97,98].

### 4.2. Randomized Controlled Trials

Several RCTs and meta-analyses of RCTs have been published on vitamin D supplementation during pregnancy and its effect on neonatal and maternal outcomes [99,100,101,102,103,104,105,106,107,108,109,110,111,112,113,114,115,116,117,118]. Amongst the many meta-analyses published, we focus on the findings of the most recent ones and/or the ones including the highest number of study participants.

Roth et al. performed a meta-analysis on vitamin D supplementation during pregnancy and maternal and neonatal/infant outcomes including the highest number of study participants [25]. They included 43 trials with 8406 participants and evaluated effects of vitamin D supplementation on 11 maternal and 27 neonatal/infant outcomes. By covering RCTs with a wide range of vitamin D doses (median dose: 2000 IU daily; range: 200 IU to 7542 IU daily) and substantial clinical and methodological heterogeneity, the authors reported that there was no evidence of any specific harm to mothers or fetuses attributable to vitamin D supplementation [25]. Due to frequently missing data on clinical outcomes, most analyses were based on only a minority of trials. Apart from an expected increase in serum/cord 25(OH)D concentrations, vitamin D supplementation increased mean birth weight by 58.33 g (95% CI: 18.88 to 97.98 g; *n* = 5273; 30 trials; *I*^2^ 43%), reduced the risk of SGA age with a pooled risk ratio of 0.60 (95% CI: 0.40 to 0.90; *n* = 741; 7 trials; *I*^2^ 0%) and reduced the risk of asthma or recurrent/persistent wheeze in the offspring by 3 years of age with a pooled risk ratio of 0.81 (95% CI: 0.67 to 0.98; *n* = 1387, 2 trials; *I*^2^ 0%). Meta-analyses on other outcomes such as preterm birth, pre-eclampsia, neonatal death, caesarian section, preterm labor or infections showed no significant effects of vitamin D. Regarding gestational diabetes mellitus, the pooled risk ratio was 0.65 (95% CI: 0.39 to 1.08; *n* = 1030; 5 trials; *I*^2^ 45%) and thus not significant, but when including all trials irrespective of minimum criteria for case definitions and methods of ascertainment the pooled risk ratio became significant with 0.61 (95% CI: 0.45 to 0.83, *n* = 2643). Roth et al. concluded that most trials were small and of low quality and that the evidence to date seems insufficient to guide clinical or policy recommendations [25].

Another recent meta-analysis by Bi et al. evaluated only outcomes in the offsprings and included 24 trials involving 5405 participants [26]. Vitamin D supplementation decreased the risk of SGA with a RR of 0.72 (95% CI: 0.52 to 0.99; *n* = 898; 6 trials; *I*^2^ 0%), increased birth weight by 75.38 g (95% CI: 22.88 to 127.88; *n* = 4087; 17 trials; *I*^2^ 44%) but had no significant effect on fetal or neonatal mortality with a RR of 0.72 (95% CI: 0.47 to 1.11; *n* = 3780; 10 trials; *I*^2^ 0%). Subgroup analyses, however, showed reduced mortality with vitamin D supplementation at a dose of ≤2000 IU daily (RR: 0.35; 95% CI: 0.15 to 0.80), but no significant effect at doses higher than 2000 IU daily (RR: 0.95; 95% CI: 0.59 to 1.54). Importantly, vitamin D supplementation was also absolutely safe in that meta-analysis. Data from only 2 RCTs showed significantly greater skinfold thickness, and higher height and weight at 3, 6, 9 and 12 months of age. In addition, vitamin D supplementation increased serum calcium levels by 0.19 mg/dL (95% CI: 0.003 to 0.38 mg/dL; *n* = 1007; 9 trials; *I*^2^ 74%) and increased Apgar scores at 1 min by 0.09 (95% CI: 0.01 to 0.17; *n* = 670; 4 trials; *I*^2^ 40%) and at 5 min by 0.08 (95% CI: 0.02 to 0.14; *n* = 668; 4 trials; *I*^2^ 13%). There were no significant effects on other outcomes such as preterm birth, infections, eczema or allergies. Specifically, there was no vitamin D effect on asthma in the infants with a RR of 0.63 (95% CI: 0.36 to 1.11; *n* = 1591; 3 trials, *I*^2^ 71%). Bi et al. conclude that vitamin D supplementation during pregnancy decreases the risk of SGA, improves infant growth and may reduce the risk of fetal or neonatal mortality at doses of ≤2000 IU daily [26].

More recently, Roth et al. published a large RCT on vitamin D supplementation during pregnancy and lactation in Bangladesh [117]. They enrolled 1300 pregnant women from 17 to 24 weeks of gestation and allocated them to placebo or weekly vitamin D doses of 4200 IU, 16,800 IU and 28,000 IU, respectively, until delivery. One additional group was allocated to weekly vitamin D doses of 28,000 IU until delivery plus further weekly doses of 28,000 IU of vitamin D for 26 weeks post partum. The main outcome was infant growth at 1 year of age, and 1164 infants were analyzed. There was no effect on any measure of infant growth at 1 year of age nor on other measures such as birth weight, SGA, preterm birth, gestational hypertension, general morbidity or mortality [117]. As expected, 25(OH)D was significantly increased by vitamin D supplementation as was serum calcium, but there was no safety concern [117]. The study by Roth et al. has to be highlighted as it is one of the few RCTs to explore outcomes according to different doses of vitamin D. Anyway, this RCT does not support the use of vitamin D supplementation in pregnancy and particularly questions whether vitamin D supplementation has any effect on infant growth. Although there were too few cases for statistical analysis it is important to note that, despite supplementation of 500 mg calcium in all study participants, there were 3 cases of rickets in the placebo group, one in the 4200 IU group but none in the groups with higher doses of vitamin D supplementation [117]. Nevertheless, Roth et al. concluded that their findings argue against routine vitamin D supplementation during pregnancy, even in communities where vitamin D deficiency is endemic. We are of the opinion that this conclusion should be discussed with a broader view on general vitamin D requirements. Major nutritional guidelines consider serum 25(OH) concentrations below 30 nmol/L as vitamin D deficient with risk of adverse skeletal health outcomes including rickets and osteomalacia. In the RCT by Roth and colleagues, the mean (±SD) serum 25(OH)D concentration of pregnant women was 27.5 (±14.2) nmol/L at baseline [117]. In the placebo group, the venous cord 25(OH)D concentration was below 30 nmol/L in 96 out of 98 analyzed samples [117]. This high prevalence of vitamin D deficiency in Bangladesh may require actions such as systematic vitamin D food fortification or routine vitamin D supplementation if we aim to meet vitamin D requirements of nutritional guidelines.

Regarding the inconsistent effects of vitamin D on asthma/wheeze in the meta-analyses by Roth et al. and Bi et al., it should be noted that a recent systematic review and meta-analysis did not examine asthma/wheeze as the comparators and outcome definitions were considered heterogeneous, whereas another meta-analysis using the same study data as Roth et al. confirmed that vitamin D reduced asthma/recurrent wheeze at the age of 3 years [80,108]. Regarding pre-eclampsia, two recent meta-analyses suggest that vitamin D reduces the RR by 0.41 (95% CI: 0.22 to 0.78) and 0.47 (95% CI: 0.24 to 0.89), respectively, and two RCTs published in 2018 and thus not included in previous meta-analyses showed or suggested reduced risk of pre-eclampsia with vitamin D supplementation in pregnancy [106,107,111,112]. By contrast, a recent Mendelian Randomization study using SNPs in genes associated with vitamin D synthesis and metabolism as exposures, argues against an effect of vitamin D on pregnancy related hypertensive disorders [116]. Thus, there are also inconsistent data on vitamin D supplementation and pre-eclampsia with some RCTs and meta-analyses suggesting a beneficial effect and others not. Regarding other outcomes such as preterm birth or gestational diabetes mellitus there are also inconsistent data from meta-analyses with some suggesting a beneficial effect of vitamin D and others do not report significant results [25,26,27,28,29,31,75]. Interestingly and in contrast to the non-pregnant state with a largely 25(OH)D independent 1,25(OH)2D production, it has been shown in vitamin D RCTs in pregnancy that production of 1,25(OH)2D was substrate dependent up to a serum 25(OH)D concentration of 100 nmol/L [35,36,38]. These findings lead, along with some observational data on 25(OH)D and adverse clinical pregnancy outcomes, to the hypothesis that vitamin D requirements in pregnancy may only be met at a serum 25(OH)D concentration of at least 100 nmol/L, a level that is much higher than currently recommended target 25(OH)D levels of nutritional vitamin D guidelines of at least 25 to 50 nmol/L [35].

### 4.3. Summary of Clinical Data on Pregnancy Outcomes

When summarizing the available evidence derived from RCTs on vitamin D supplementation in pregnancy we conclude that vitamin D is safe and improves vitamin D and calcium status, thereby protecting skeletal health. Data of RCTs and meta-analyses of RCTs suggest some other beneficial effects but are inconsistent on whether vitamin D supplementation improves clinical neonatal or maternal outcomes such as SGA, fetal/infant growth, infant/neonatal mortality, asthma/wheeze, pre-eclampsia or gestational diabetes mellitus. However, it must be stressed that for most of these outcomes the evidence is at least numerically and, by trend, in favour of beneficial vitamin D effects. Furthermore, when some vitamin D RCTs with inconclusive results on an intention to treat basis were re-analyzed by achieved serum 25(OH)D concentrations as a marker of treatment adherence, some beneficial vitamin D effects became statistically significant [35]. Nevertheless, while inconsistent results of meta-analyses can partially be attributed to methodological issues such as study selection, it must be recognized that we do not have sufficient RCT data on potential effects of vitamin D supplementation during the first weeks of pregnancy. These first weeks of pregnancy are, however, known to be very crucial and sensitive to deficiencies of other micronutrients such as folate or to hormone deficiencies such as hypothyroidism. RCTs on preconception vitamin D supplementation are therefore needed.

## 5. Lactation

There are several studies that addressed the vitamin D content of breast milk and the effect of maternal vitamin D supplementation on vitamin D and 25(OH)D concentration in breast milk [119,120,121,122,123,124,125,126]. Vitamin D content of breast milk is often expressed as antirachitic activity (ARA), i.e., the ARA of the sum of vitamin D plus 25(OH)D. Assuming an average milk intake of 780 ml per day, the ARA of breast milk has to be 513 IU/L to provide the same vitamin D supply for infants as a daily vitamin D supplement with 400 IU of vitamin D, that is usually recommended for infants for the purpose of rickets prevention [126]. However, it should be noted that only minimal amounts of maternal serum 25(OH)D are transferred to human breast milk, and vitamin D concentration in breast milk is only approximately as high as 20% of the maternal serum vitamin D concentrations. Therefore, to provide sufficient vitamin D content in breast milk for the infant, the vitamin D intake of the mother during lactation has to be much higher compared to the intake during pregnancy as at birth, serum 25(OH)D concentrations in cord blood are about 50 to 80% of serum 25(OH)D concentrations of the mother [39].

A survey in different countries on ARA of breast milk reported a median concentration of 45 IU/L with no sample having a concentration of 513 IU/L that would equal an infant vitamin D supplement intake of 400 IU per day [126]. In a RCT by Hollis et al., exclusively breastfeeding women at 4 to 6 weeks postpartum were randomized to 400, 2400 or 6400 IU of vitamin D per day for 6 months with additional vitamin D supplementation of 400 IU per day of the infants of mothers allocated to 400 IU, but only placebo and, therefore, no infant vitamin D supplementation in the other two groups [120]. Main outcome was that infants, who did not receive any vitamin D supplement but whose mothers were allocated to 6400 IU of vitamin D per day, achieved the same sufficient vitamin D status compared to infants, who received themselves a daily vitamin D supplement containing 400 IU [120]. Importantly, due to insufficient effects of 2400 IU of vitamin D on breast milk content of infants with subsequent safety concerns, the 2400 IU group was stopped by the data safety monitoring board. Thus, adequate vitamin D supply by breast milk is possible but can only be achieved by relatively high doses of maternal vitamin D supplementation. Considering that vitamin D has a half-life in serum of only about a day, it has thus been suggested that breastfeeding women should prefer daily over intermittent vitamin D supplementation to increase the vitamin D content in their breast milk. In a RCT, Oberhalm et al., however, observed that when comparing a single vitamin D dose of 150,000 IU compared to daily intake of 5000 IU for 28 days in breastfeeding women, the serum 25(OH)D concentrations in the infants were similar at study end [121]. Anyway, considering that vitamin D itself may hypothetically exert some non-genomic effects and has a short half-life, and taking into account results from some RCTs in the elderly suggesting that intermittent high-dose vitamin D supplementation may even be harmful, it may be argued that daily vitamin D supplementation should be preferred [3,35]. Nevertheless, effects of vitamin D supplementation of lactating women on clinical outcomes in the infants are still not well investigated.

## 6. Clinical Considerations

We wish to emphasize that inadequate vitamin D intakes and vitamin D deficiency in pregnant and lactating women are very common worldwide, thus pointing to the need to improve vitamin D status [127,128,129,130,131,132,133,134,135,136,137,138,139,140,141,142,143]. A systematic review has shown that 25(OH)D concentrations below 50 and 25 nmol/L during pregnancy were reported in 64% and 9% of Americans, and 57% and 23% of Europeans [127]. The prevalence of vitamin D deficiency is particularly high during winter. A Germany study showed that 67% of pregnant women had serum 25(OH)D concentrations below 25 nmol/L in this season [132]. Similar findings have been reported in cord blood samples in Germany [142]. Furthermore, women planning pregnancy have also a high prevalence of vitamin D deficiency, and it should be considered that hormonal contraceptives, that are taken by approximately every second women in the reproductive age in Western countries, increase serum DBP and thus total 25(OH)D concentrations (by about 26%) but have no effect on free 25(OH)D [140]. Therefore, we might overestimate vitamin D status by measuring total 25(OH)D in women taking hormonal contraceptives and, consequently, total 25(OH)D will likewise decrease after stopping hormonal contraception [140]. Although it is currently not considered in clinical routine, it should be noted that certain single nucleotide polymorphisms (SNPs) determine a few percent of the variation in 25(OH)D concentrations and our response to vitamin D supplementation whereas vitamin D itself may impact on epigenetics [143,144,145]. Interestingly, it has been hypothesized that vitamin D might exert epigenetic effects in utero and early life, i.e., vitamin D modifies gene expression without changes in the DNA code itself that may improve early development and later health [138].

The burning question is, of course, how to deal in clinical routine with vitamin D treatment during pregnancy and lactation. There is no clear answer to this question but following nutritional guidelines it is usually recommended to achieve vitamin D intakes ranging from 400 to 800 IU of vitamin D per day in order to reach serum 25(OH)D target concentrations of at least 25 to 50 nmol/L [137]. In pregnancy, however, it should be considered that cord blood 25(OH)D concentrations are only about 50 to 80% of serum 25(OH)D concentrations. Therefore, it could be hypothesized, that the mother may need more 25(OH)D during pregnancy in order to transfer enough 25(OH)D to the fetus [39]. In this context, a recent RCT in Ireland has shown that an overall vitamin D intake (supplemental plus nutritional vitamin D) of almost 1200 IU of vitamin D per day was required to ensure that cord serum 25(OH)D concentrations were above 30 nmol/L in 95%, and above 25 nmol/L in 99% of the infants, respectively [39]. Considering that the mean dietary vitamin D intake is usually not higher than 200 IU per day in many countries a supplemental vitamin D intake of about 1000 IU per day would be necessary to ensure a sufficient vitamin D supply. Nevertheless, some experts argue that whereas relatively low vitamin D intakes are sufficient for musculoskeletal health, the vitamin D requirements may be much higher for protection of extraskeletal health outcomes in pregnancy [35,36,38]. Therefore, some vitamin D supplementation guidelines recommend for pregnant women and women planning pregnancy a daily vitamin D intake of 1500–2000 IU in order to obtain and maintain 25(OH)D concentrations as high as 75 nmol/L [146]. However, it is not clearly proven that serum 25(OH)D concentrations of ≥75 nmol/L provide additional health benefits over levels of ≥50 nmol/L. Whether such high or even higher vitamin D doses exert additional beneficial effects on extraskeletal health outcomes is currently evaluated in several vitamin D RCTs in pregnancy [25]. Nevertheless, it should be considered that several vitamin D RCTs are limited by not fully considering some issues that are relevant for RCTs on nutrients such as dose response curves, inclusion of study participants with vitamin D deficiency [147,148,149]. Furthermore, future studies should aim to address several knowledge gaps such as data on vitamin D supplementation starting preconceptional or very early in pregnancy, more data on vitamin D requirements in the first year of life, more data on interactions of vitamin D with PTH and other parameters of mineral metabolism. Future RCTs should always include standardized serum 25(OH)D measurements at baseline and follow-up, clear definitions of clinical outcomes and assessments of additional calcium intake because vitamin D requirements may be different depending on intakes of calcium. Furthermore, an international registry of rickets has been proposed as an important goal for future research activities [150].

## 7. Conclusions

Current evidence from clinical studies indicates that vitamin D deficiency is very common in pregnant and lactating women and is associated with a variety of adverse outcomes. By contrast, data from RCTs have yielded inconsistent results. There may be some beneficial effects of vitamin D supplementation on clinical pregnancy outcomes, but it is premature to draw final conclusions and there are only insufficient RCT data on the potential effects of vitamin D in the first weeks of pregnancy. Importantly, it is clearly established that vitamin D supplementation at commonly used doses in pregnant and lactating women is safe and can effectively improve the vitamin D and calcium status of the fetus and infant, thereby protecting skeletal health. Current guidelines recommend a vitamin D intake of 400 to 800 IU per day for pregnant women. Our conclusion is that when a woman wants to ensure a sufficient vitamin D supply to their fetus or infant, an intake of a vitamin D supplement at a dose of 800 to 1000 IU per day during preconception or pregnancy is sufficient to achieve serum 25(OH)D target concentrations as recommended by vitamin D guidelines.

## Figures and Tables

**Table 1:** Dietary reference values (DRV)/dietary reference intakes (DRI) for vitamin D.

Country (Health Authority)	USA and Canada (IOM)		Europe (EFSA)	Germany, Austria and Switzerland (DACH)	UK (SACN)	Nordic European Countries (NORDEN)
DRV/DRI	EAR	RDA	AI	AI	RNI	RI
Target 25(OH)D in nmol/L	40	50	50	50	25	50
Age group	Vitamin D intakes international units per day (40 international units = 1 μg)					
0–6 months	400			400	340 to 400	
7–12 months	400		400	400	340 to 400	400
1–3 years	400	600	600	800	400	400
4–6 years	400	600	600	800	400	400
7–8 years	400	600	600	800	400	400
9–10 years	400	600	600	800	400	400
11–14 years	400	600	600	800	400	400
15–17 years	400	600	600	800	400	400
18–69 years	400	600	600	800	400	400
70–74 years	400	800	600	800	400	400
75 years and older	400	800	600	800	400	800
Pregnancy	400	600	600	800	400	400
Lactation	400	600	600	800	400	400

IOM, Institute of Medicine; EFSA, European Food Safety Authority; DACH, Germany, Austria and Switzerland; SACN, Scientific Advisory Committee on Nutrition; EAR, estimated average requirement. RDA, recommended dietary allowance; AI, adequate intake; RNI, reference nutrient intake; RI, recommended intake; 25(OH)D, 25-hydroxyvitamin D; table adopted and modified from [3].
